# Effect of Surface Charge Characteristics of Ferroelectric LiNbO_3_ on Wettability of Ionic Liquids

**DOI:** 10.3390/nano12122085

**Published:** 2022-06-17

**Authors:** Bo Tang, Yiwen Zhao, Sen Yang, Zhiang Guo, Zhenhui Wang, An Xing, Xiaoyan Liu

**Affiliations:** Chongqing Key Laboratory of Nano/Micro Composites and Devices, College of Metallurgy and Materials Engineering, Chongqing University of Science and Technology, Chongqing 401331, China; tb19971209@163.com (B.T.); zyw13989150751@163.com (Y.Z.); yangsen20210510@163.com (S.Y.); 13705292022@163.com (Z.G.); 15061688265@163.com (Z.W.); devid14@163.com (A.X.)

**Keywords:** ferroelectrics, surface charge characteristics, interfaces, ionic liquids, wettability

## Abstract

Electrowetting is a widely used and effective method to tune the wettability of ionic liquids at solid-liquid interfaces, but it usually requires an external electric field. Here, we proposed a strategy for conveniently tuning ionic liquid wettability by adopting ferroelectric LiNbO_3_ single crystals as functional substrates. A heating pretreatment process was applied to modulate the surface charge characteristics of LiNbO_3_ substrates, leading to an improved wettability of [EMIM][BF_4_] and [EMIM][NTf_2_] on the LiNbO_3_ substrates with both positively poled (+Z) and negatively poled (−Z) surfaces. This work may be of great interest in the field of ferroelectric-based microelectronics.

## 1. Introduction

Surface wettability plays a vital role in numerous engineering fields, such as superhydrophobic surfaces in windshields, superhydrophilic surfaces in microelectronics and self-cleaning surfaces. The main factors affecting surface wettability are surface chemistry and surface structure [1]. External factors such as light, electricity, magnetism and heat also have a great impact on surface wettability [2].

Ionic liquids (ILs) are also known as room-temperature melt salts, usually composed of organic cations and inorganic/organic anions. A wide variety of ILs can be designed by combining different anions and cations. As a “green solvent”, ILs have been used in fields of chemical research including catalysis [3,4], separation [5,6], and electrochemistry [7,8] due to their unique properties of low vapor pressure, strong solubility, excellent thermal stability, and a wide electrochemical window [9]. Most physical and chemical reactions in the applications occur on the solid-liquid interface [10]. For example, the ionic liquid (IL) wettability on catalyst surfaces greatly affects catalytic efficiency and that occurring on electrode surfaces determines the final performance of photoelectrochemical devices.

Since the electrostatic force between anions/cations of ILs and charges of solid surfaces can increase the wetting tension (*W*_el_) and reduce the contact angle (*θ*), the charge distribution on the solid surface can significantly affect the wettability of ILs. Surface chemical modification and electrowetting are the main methods to modulate the charge distribution on the solid surface. Surface chemical modification refers to the modification of specific electrochemical groups on the solid surface to change the charge distribution, thereby adjusting the wettability of ILs on the solid surface [11,12,13]. Li et al. [13] used chemical grafting to precisely control the surface charge of the porous network of poly (tert-butyl acrylate) (PtBA) to adjust the wettability of specific ILs and prepare functional membrane materials for the efficient recovery of ILs. Electrowetting refers to adjusting the wettability of ILs by applying an external electric field to change the charge distribution at the solid-liquid interface. [14,15,16,17,18]. Li et al. [16] placed a conductive polymer with properties similar to ILs on a low surface energy-treated SiO_2_/Si substrate and adjusted the interaction between solid-liquid interface charges to reduce the saturation contact angle by changing the polarity and magnitude of the applied voltage. Restolho et al. [17] found that ILs have a similar electrowetting behavior to traditional salt solutions on the hydrophobic surface and that the contact angle of ILs decreases with the increase of the applied voltage. Liu et al. [18] studied the electrowetting characteristics of imidazole-based ILs and found that the wettability of ILs on the negatively charged surface of the electrode plate was more significant.

Ferroelectric materials have a net electric dipole moment in the absence of an external field due to the non-coinciding center of the positive and negative charges in the crystal lattice, which is known as spontaneous polarization (*P*). The polarity of spontaneous polarization can be reversed by applying an external electric field beyond the intrinsic coercive field (*E*_c_). The LiNbO_3_ single crystal is a well-known ferroelectric material and has found a variety of applications in electro-optics and non-linear optics [19,20]. Below the Curie temperature (*T*_c_~1210 °C), the Li and Nb ions in the LN single crystal move along the c-axis to form a large spontaneous polarization of about 75 μC/cm^2^ [21]. The large spontaneous polarization generates numerous bound charges on the LiNbO_3_ inner surface perpendicular to the crystallographic c-axis, i.e., *σ*_pol_ = *Pn*, where *σ*_pol_ is the polarization bound charges density and *n* is the normal unit vector. The surface accumulating with positive bound charges is referred to as a positively poled surface (+Z surface), while that with negative bound charges is noted as a negatively poled surface (−Z surface). Therefore, the LiNbO_3_ single crystal with +Z and −Z surfaces can exhibit strong surface charge characteristics and is suitable for use as a functional substrate [22,23,24,25,26,27,28,29,30]. At room temperature (RT) in air, the polarization bound charges are usually neutralized by charged particles absorbed from the environment [31,32,33,34]. However, the surface neutralization can be disrupted with a thermal stimulation due to the pyroelectric effect, i.e., d*P* = *ρ*d*T*, where *ρ* is the pyroelectric coefficient and d*T* is the temperature change.

In this work, LiNbO_3_ single crystals with +Z and −Z surfaces are used as the functional substrates, while a glass slide is used as the control substrate. 1-ethyl-3-methylimidazolium bis[trifluoromethylsulfonyl]imide ([EMIM][NTf_2_]) and 1-ethyl-3-methylimidazolium tetrafluoroborate ([EMIM][BF_4_]) are selected to investigate the influence of the surface tensions of different ILs species on the wettability. A heating pretreatment with a temperature of up to 110 °C was conducted to modulate the charge distribution of the LiNbO_3_ substrates. Since the heating temperature (110 °C) is far below the Curie temperature (~1210 °C), the heating pretreatment will not affect the surface charge characteristics of the LiNbO_3_ substrate. The effect of the solid surface charge and IL surface tensions on the wettability was analyzed by comparing the contact angle of the ILs on the LiNbO_3_ and glass substrates going through the same heating pretreatment process. Different from surface chemical modification and electrowetting, the surface charge distribution of ferroelectric LiNbO_3_ can be simply modulated by the heating pretreatment. However, further studies are needed to enhance the wettability variation of ILs on ferroelectric substrates.

## 2. Materials and Methods

Both [EMIM][NTf_2_] and [EMIM][BF_4_] with a purity of >90% were purchased from Macklin Biochemical Co., Ltd. (Shanghai, China). The surface tensions (RT in air) of [EMIM][NTf_2_] and [EMIM][BF_4_] are ~36 mN/m and ~49 mN/m, respectively [17]. LiNbO_3_ single crystals were purchased from Yamaju Ceramics Co., Ltd. (Nagoya, Japan), and glass slides were purchased from Feizhou Glass Co., Ltd. (Yancheng, China). The size of all the substrates was 1 × 1 cm^2^. All the substrates were ultrasonically cleaned with acetone, ethanol and deionized water to remove surface contaminants, and then blow-dried with nitrogen. A contact angle meter (SIND-200T, Shengding Precision Instrument Co., Ltd., Dongguan, China) was used to measure the contact angle of the ILs on the substrates before and after the heating pretreatment. The surface potential property of the LiNbO_3_ substrates before and after the heating pretreatment was characterized by Kelvin probe force microscopy (KPFM) via scanning a tip at a constant level of 50 nm from the substrate surface at a fast scan rate of 2 Hz. The surface morphology of the substrates used in this work was characterized by an atomic force microscope (AFM) in the ac mode. Both KPFM and AFM measurements were performed through a scanning probe microscope (Cypher S, Oxford Instrument Technology Co., Ltd., Oxford, UK) by using a conductive cantilever with a spring constant of 2.8 N/m.

The heating pretreatment process is as follows: first set a heating rate of 5 °C/min by tuning the power of the temperature control station (PR-RE-TC, Purui Material Technology Co., Ltd., Shenzhen, China), then heat the substrate to 110 °C and keep it for 10 min, and finally cool down rapidly to the RT. A 1 μL droplet was immediately added to the center of the substrate, and the contact angle was measured when the droplet was stable. The average of three contact angle values measured from each substrate was adopted.

## 3. Results

**Figure 1:**
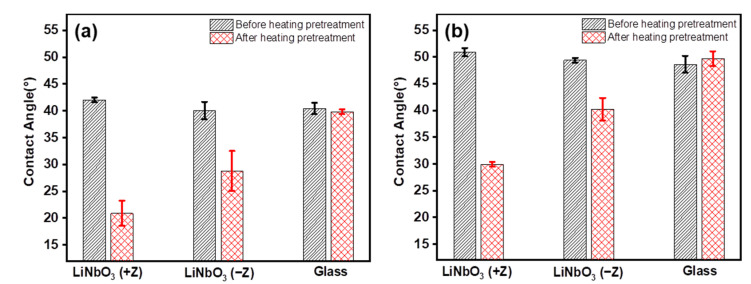
Contact angle of the ionic liquids (ILs) on the LiNbO_3_ and glass substrates before and after the heating pretreatment. (**a**) [EMIM][NTf_2_], (**b**) [EMIM][BF_4_].

Figure 1 shows the contact angle of [EMIM][NTf_2_] and [EMIM][BF_4_] on the +Z and −Z surfaces of the LiNbO_3_ and glass substrates before and after the heating pretreatment. It can be seen that the contact angles of both the ILs on the +Z and −Z surfaces of the LiNbO_3_ substrate are obviously different before and after the heating pretreatment, while the difference of both the ILs on the glass substrate is limited. The contact angles of [EMIM][BF_4_] with higher surface tensions are larger than those of [EMIM][NTf_2_] on all the substrates before and after the heating pretreatment. In addition, after the heating pretreatment, the contact angles of the ILs on both the +Z and −Z surfaces of the LiNbO_3_ substrate are reduced, and the contact angles of the ILs on the +Z surface are reduced to a greater extent than those on the −Z surface.

**Figure 2:**
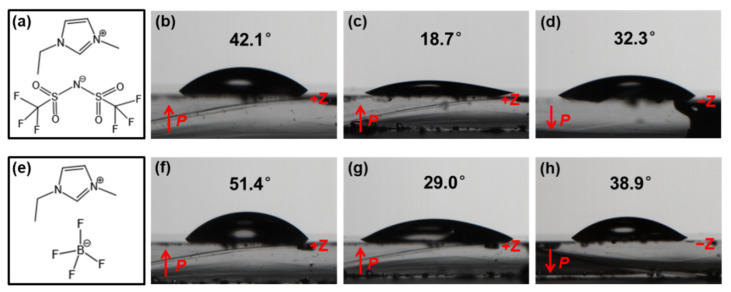
Contact angle of [EMIM][NTf_2_] and [EMIM][BF_4_] on the LiNbO_3_ substrate (**b**,**f**) before the heating pretreatment with the +Z surface and after the heating pretreatment with (**c**,**g**) the +Z surface and (**d**,**h**) −Z surface. (**a**,**e**) Chemical structure of [EMIM][NTf_2_] and [EMIM][BF_4_].

Figure 2 illustrates the effect of the polarization orientation, the heating pretreatment, and the anions on the contact angle of the ILs with the LiNbO_3_ substrate. After the heating pretreatment of the LiNbO_3_ substrate, the contact angle of both [EMIM][NTf_2_] and [EMIM][BF_4_] decreased with either the +Z surface or the −Z surface. Compared with the different polarization orientation of the LiNbO_3_ substrate after the heating pretreatment, the contact angles of both the ILs on the +Z surface are smaller than those on the −Z surface. Overall, [EMIM][BF_4_] presents larger contact angles than [EMIM][NTf_2_].

**Figure 3:**
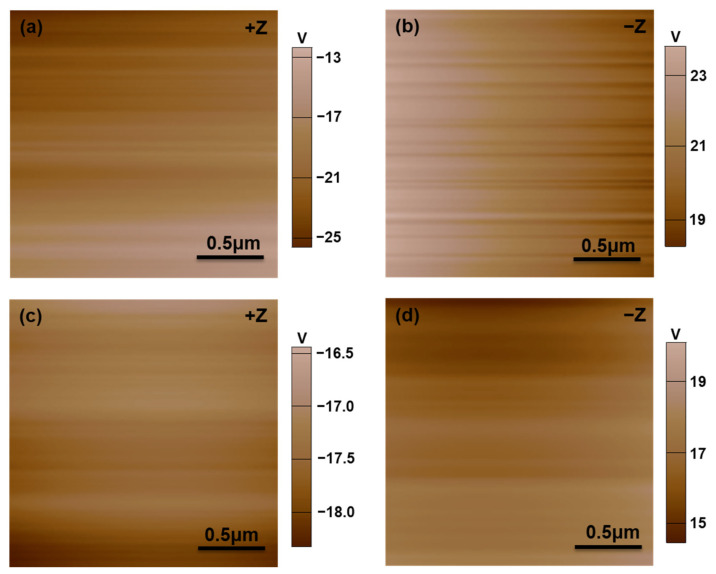
KPFM surface potential images of the LiNbO_3_ substrates (**a**,**b**) before the heating pretreatment and (**c**,**d**) right after the heating pretreatment.

The surface charge characteristics of the LiNbO_3_ substrates before and right after the heating pretreatment were investigated by KPFM. As shown in Figure 3, the LiNbO_3_ substrates exhibit a larger average surface potential before the heating pretreatment (Figure 3a,b) than after the heating pretreatment (Figure 3c,d), with either the +Z surface or −Z surface.

**Figure 4:**
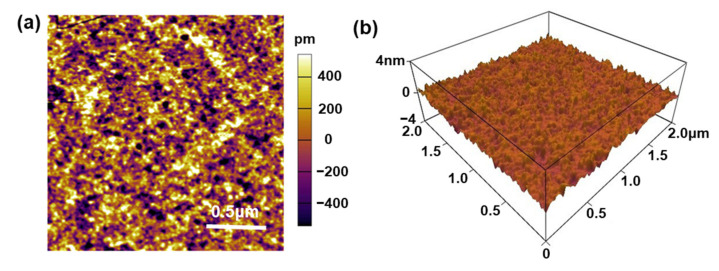
(**a**) Representative AFM topography image and (**b**) corresponding 3D surface plot of the LiNbO_3_ substrate.

In order to exclude the influence of the surface microstructure on the wettability of ILs, An ac-AFM was used to characterize the surface morphology of the LiNbO_3_ and glass substrates. Figure 4 is a representative AFM topography image and corresponding 3D surface plot of the LiNbO_3_ substrate, demonstrating that the surface average roughness is about 0.2 nm. Our AFM results found no significant difference in surface morphology between the LiNbO_3_ and glass substrates before and after the heating pretreatment. Since the main factors affecting surface wettability are surface chemistry and surface structure, we believe that differences in the IL wettability on different substrates used in this work are mainly attributable to surface charge characteristics.

## 4. Discussion

The LiNbO_3_ single crystal possesses a large spontaneous polarization (75 μC/cm^2^) that generates a large number of polarization bound charges (*σ*_pol_). At RT in air, the bound charges are always screened by oppositely charged particles absorbing from the environment. Screening charges (*σ*_s_) are considered to be composed of accumulating charges located in tightly adsorbed layers adjacent to the surface and loosely packed layers upon them. Partial screening charges can be removed by heating the substrate to a temperature above 100 °C [31,33]. Our KPFM measurement results (Figure 3) also support this assumption. Both the +Z and −Z surfaces of the LiNbO_3_ substrate exhibit a larger average surface potential before the heating pretreatment (Figure 3a,b) than after the heating pretreatment because some screening charges were removed by heating the substrate up to 110 °C and a re-screening process cannot be fully completed in a short period of time.

**Figure 5:**
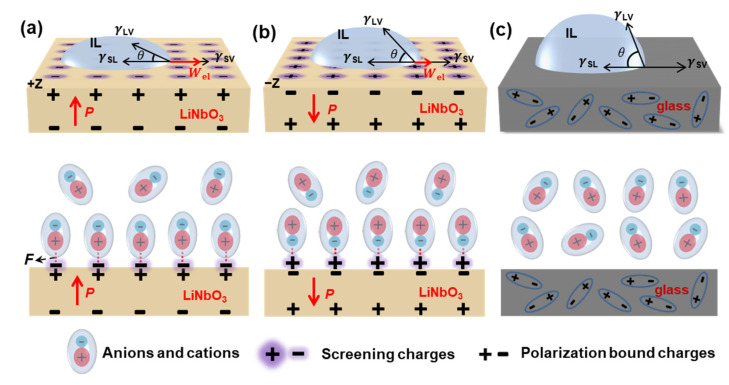
Wetting mechanisms of the IL on the LiNbO_3_ and glass substrates after the heating pretreatment. (**a**) LiNbO_3_ substrate with +Z surface, (**b**) LiNbO_3_ substrate with −Z surface, (**c**) glass substrate.

Figure 5 shows the wetting mechanism of the ILs on the +Z and −Z surfaces of the LiNbO_3_ and glass substrates after the heating pretreatment, respectively. In the case of the LiNbO_3_ substrates, some screening charges can be removed after the heating pretreatment. When dropping an IL droplet immediately after the heating pretreatment onto the substrate, the anions and cations of the ILs interact with the screening charges in the tightly adsorbed layers on the LiNbO_3_ substrate by an electrostatic Coulomb force (*F*), resulting in a larger wetting tension. In general, the actual contact angle can be expressed as below [13]:

$$\cos\theta =\frac{\gamma_{\mathrm{SV}}-\gamma_{\mathrm{SL}}+W_{\mathrm{el}}}{\gamma_{\mathrm{LV}}}$$

where *θ* is the actual contact angle, $W_{el}$ is the wetting tension, and $\gamma_{SV}$, $\gamma_{SL}$ and $\gamma_{LV}$ refer to the interfacial tensions of solid-gas, solid-liquid and gas-liquid interfaces, respectively. After the heating pretreatment, the ILs on the LiNbO_3_ substrates have larger wetting tensions, and thus smaller contact angles (Figure 1).

Furthermore, the ILs exhibited different contact angles on the +Z and −Z surfaces of the LiNbO_3_ substrates after the heating pretreatment, i.e., *θ*_(+Z)_ < *θ*_(−Z)_. Since the volume of cations is larger than that of anions, the electrostatic Coulomb forces between anions/+Z surface and cations/−Z surface are different, i.e., *F*_(+Z)_ > *F*_(−Z)_. Therefore, after the heating pretreatment, the wetting tensions of the ILs on the +Z surface are larger than those of the ILs on the −Z surface, leading to a smaller contact angle and better wettability on the +Z surface of the LiNbO_3_ substrate. In addition, when comparing the different ILs used in this work, the overall wettability of [EMIM][NTf_2_] (Figure 1a) is better than that of [EMIM][BF_4_] (Figure 1b). [EMIM][NTf_2_] has a smaller surface tension [17], resulting in a smaller contact angle and thus a better wettability.

Glass does not have spontaneous polarization properties. The surface charge distribution of glass is not significantly affected by temperature due to a random arrangement of dipoles in glass, resulting in slight differences in the ILs’ wettability on the glass substrate before and after the heating pretreatment (Figure 1).

## 5. Conclusions

In summary, this work reports the effect of the surface charge characteristics of ferroelectric LiNbO_3_ on IL wettability at the solid-liquid interfaces. The surface charge neutralization of LiNbO_3_ was destructed by the heating pretreatment. The wettability of [EMIM][BF_4_] and [EMIM][NTf_2_] on LiNbO_3_ substrates with both the +Z and −Z surfaces increased after the heating pretreatment due to the interaction between anions/cations of the ILs and surface charges of the LiNbO_3_ substrates. The difference in wettability observed between [EMIM][BF_4_] and [EMIM][NTf_2_] is attributed to their different surface tensions. This finding can be used to facilitate fluid transport in ferroelectric-based microfluidic applications.

## Data Availability

Not applicable.

## References

[1] Gao J., Zhao J., Liu L., Xue W. (2016). Dimensional effects of polymer pillar arrays on hydrophobicity. Surf. Eng..

[2] Kang Z., Wang S., Fa N., Xiao Z., Wang R., Sun D. (2016). Surface wettability switching of metal-organic framework mesh for oil-water separation. Mater. Lett..

[3] Hecht D.S., Hu L., Irvin G. (2011). Emerging transparent electrodes based on thin films of carbon nanotubes, graphene, and metallic nanostructures. Adv. Mater..

[4] Duan Y., Tang Q., Liu J., He B., Yu L. (2014). Transparent metal selenide alloy counter electrodes for high-efficiency bifacial dye-sensitized solar cells. Angew. Chem. Int. Edit..

[5] Bae S., Kim H., Lee Y., Xu X., Park J., Zheng Y., Balakrishnan J., Lei T., Ri Kim H., Song Y.I. (2010). Roll-to-roll production of 30-inch graphene films for transparent electrodes. Nat. Nanotechnol..

[6] Kim A., Won Y., Woo K., Kim C., Moon J. (2013). Highly transparent low resistance ZnO/Ag nanowire/ZnO composite electrode for thin film solar cells. ACS Nano.

[7] Wang W., Song M., Bae T., Park Y.H., Kang Y., Lee S., Kim S., Kim D.H., Lee S., Min G. (2014). Transparent ultrathin oxygen-doped silver electrodes for flexible organic solar cells. Adv. Funct. Mater..

[8] Zhang C., Zhao D., Gu D., Kim H., Ling T., Wu Y.R., Guo L.J. (2014). An ultrathin, smooth, and low-loss Al-doped Ag film and its application as a transparent electrode in organic photovoltaics. Adv. Mater..

[9] Hallett J.P., Welton T. (2011). Room-temperature ionic liquids: Solvents for synthesis and catalysis. Chem. Rev..

[10] Liu H., Jiang L. (2016). Wettability by ionic liquids. Small.

[11] Liu H., Yi D., Zhuo A., Zhou Y., Lei J. (2015). Fabricating surfaces with tunable wettability and adhesion by ionic liquids in a wide range. Small.

[12] Zhang J., Liu H., Jiang L. (2017). G-Membrane-Based strategy for efficient ionic LiquidsWater separation assisted by superwetta-bility. Adv. Funct. Mater..

[13] Li L., Chang L., Zhang X., Liu H., Jiang L. (2017). Surface charge-induced efficient recovery of ionic liquids from aqueous phase. ACS Appl. Mater. Inter..

[14] Amrouche F., Gomari S.R., Islam M., Xu D. (2019). New insights into the application of a magnetic field to enhance oil recovery from oil-wet carbonate reservoirs. Energ. Fuel..

[15] Amrouche F., Gomari S.R., Islam M., Xu D. (2020). A novel hybrid technique to enhance oil production from oil-wet carbonate reservoirs by combining a magnetic field with alumina and iron oxide nanoparticles. J. Clean. Prod..

[16] Li X., Tian H., Shao J., Ding Y., Chen X., Wang L., Lu B. (2016). Decreasing the saturated contact angle in electrowetting-on-dielectrics by controlling the charge trapping at liquid-solid interfaces. Adv. Funct. Mater..

[17] Restolho J., Mata J.L., Saramago B. (2009). Electrowetting of ionic liquids: Contact angle saturation and irreversibility. J. Phys. Chem. C..

[18] Liu Z., Cui T., Li G.Z., Endres F. (2017). Interfacial nanostructure and asymmetric electrowetting of ionic liquids. Langmuir.

[19] Zhang M., Buscaino B., Wang C., Shams-Ansari A., Reimer C., Zhu R., Kahn J.M., Lončar M. (2019). Broadband electro-optic frequency comb generation in a lithium niobate microring resonator. Nature.

[20] Wang C., Zhang M., Stern B., Lipson M., Loncar M. (2017). Nanophotonic lithium niobate electro-optic modulators. Opt. Express.

[21] Savage A. (1966). Pyroelectricity and spontaneous polarization in LiNbO_3_. J. Appl. Phys..

[22] Liu X., Zhang Q., Li J., Valanoor N., Tang X., Cao G. (2018). Increase of power conversion efficiency in dye-sensitized solar cells through ferroelectric substrate induced charge transport enhancement. Sci. Rep..

[23] Hnilova M., Liu X., Yuca E., Jia C., Wilson B., Karatas A.Y., Gresswell C., Ohuchi F., Kitamura K., Tamerler C. (2012). Multifunctional protein-enabled patterning on arrayed ferroelectric materials. ACS Appl. Mater. Inter..

[24] Liu X., Kitamura K., Yu Q., Xu J., Osada M., Takahiro N., Li J., Cao G. (2013). Tunable and highly reproducible surface-enhanced raman scattering substrates made from large-scale nanoparticle arrays based on periodically poled LiNbO_3_ templates. Sci. Technol. Adv. Mat..

[25] Liu X., Hatano H., Takekawa S., Ohuchi F., Kitamura K. (2011). Patterning of silver nanoparticles on visible light-sensitive Mn-doped lithium niobate photogalvanic crystals. Appl. Phys. Lett..

[26] Al-Shammari R.M., Baghban M.A., Alattar N., Gowen A.A., Rodriguez B.J. (2018). Photo-Induced enhanced Raman from lithium niobate on insulator template. ACS Appl. Mater. Inter..

[27] Liu X., Osada M., Kitamura K., Nagata T., Si D. (2017). Ferroelectric-assisted gold nanoparticles array for centimeter-scale highly reproducible SERS substrates. Sci. Rep..

[28] Liu X., Ohuchi F., Kitamura K. (2008). Patterning of surface electronic properties and selective silver deposition on LiNbO_3_ template. Funct. Mater. Lett..

[29] Carville N.C., Neumayer S.M., Manzo M., Gallo K., Rodriguez B.J. (2016). Biocompatible gold nanoparticle arrays photodeposited on periodically proton exchanged lithium niobate. ACS Biomater. Sci. Eng..

[30] Liu X., Kitamura K., Terabe K., Hatano H., Ohashi N. (2007). Photocatalytic nanoparticle deposition on LiNbO_3_ nanodomain patterns via photovoltaic effect. Appl. Phys. Lett..

[31] Liu X., Kitamura K., Terabe K. (2006). Surface potential imaging of nanoscale LiNbO_3_ domains investigated by electrostatic force microscopy. Appl. Phys. Lett..

[32] Neumsyer S., Levlev A.V., Collins L., Vasudevan R., Baghban M.A., Ovchinnikova O.S., Jesse S., Gallo K., Rodriguez B.J., Kalinin S.V. (2018). Surface chemistry controls anomalous ferroelectric behavior in lithium niobate. ACS Appl. Mater. Inter..

[33] Liu X., Terabe K., Kitamura K. (2006). Surface potential properties on near-stoichiometric LiNbO_3_ crystals with nanoscale domain-engineered structures. J. Electroceram..

[34] Yue C., Lu X., Zhang J., Huang F., Zhu J. (2019). Ferroelectric surface chemistry: First-principles study of adsorption on the stoichiometric LiNbO_3_ (0001) surface. Phys. Rev. B.

